# Laparoscopic Transduodenal Ampullectomy for a High-Grade Dysplasia Tumor

**DOI:** 10.7759/cureus.62761

**Published:** 2024-06-20

**Authors:** Zoi Nitsa, Prodromos Kanavidis, Spyridon Davakis, Alexandros Charalabopoulos

**Affiliations:** 1 1st Department of Surgery, University of Athens, Laiko Hospital, Athens, GRC

**Keywords:** vater, laparoscopic treatment, minimally invasive pancreatic surgery, tumors of the ampulla, transduodenal ampullectomy

## Abstract

Ampullary tumors occur rarely, and the only curative treatment is resection. The endoscopic approach is the most well-known and common treatment of choice. Open surgical resection is the usual treatment of choice in cases of unsuccessful endoscopic resection and big tumors. Although the technically challenging laparoscopic approach is not yet widespread, it is a feasible and safe method in well-selected patients. This case report focuses on the case of a 74-year-old male with high-grade dysplasia in the ampulla of Vater, who had an incomplete endoscopic resection. Therefore, we performed a laparoscopic transduodenal ampullectomy.

## Introduction

Ampullary tumors are rare, with an incidence of fewer than 0.001% per year [1]. There are two therapy options for periampullary and ampullary tumors, namely, endoscopic excision or surgical resection as pancreatoduodenectomy (PD) and transduodenal ampullectomy (TA). There are three approaches of TA, i.e., open, laparoscopic, and robotic. PD is performed for malignant ampullary tumors for which lymph node clearance is necessary (tumors T2 or higher) [2]. TA can be the surgical treatment of choice for either benign tumors or in situ neoplasms (Tis) of the ampulla that have had an incomplete resection (positive margins) or endoscopic resection is not possible due to their size or location [3]. Relative to pancreatectomy, TA is beneficial in preserving pancreatic function [2]. Resection of T1-stage tumors has been controversial, with the risk of lymph node metastasis ranging from 22% to 30% [4]. However, no clear evidence-based guidelines have been published. There are few published cases regarding the laparoscopic approach, which is a safe and effective method, even though it requires surgical skills, with more than 100 cases reported in five years. [2] This case report aims to present the technique of laparoscopic TA.

## Case presentation

A 74-year-old male with mild comorbidities (atrial fibrillation and controlled hypertension; ASA II) presented with a history of night chills and persistent low-grade evening fever for three months. Laboratory examinations and abdominal MRI revealed elevated liver function tests (gamma-glutamyl transferase = 110 U/L, alkaline phosphatase = 220 U/L, total bilirubin = 1.86 mg/dL, direct bilirubin = 1.33 mg/dL) and a 2.5 cm tumor in the ampulla of Vater. He underwent endoscopic resection and the biopsy results showed high-grade dysplasia that was not fully excised. As the residual tumor was not metabolically active on the PET/CT scan, laparoscopic TA was chosen.

**Figure 1:**
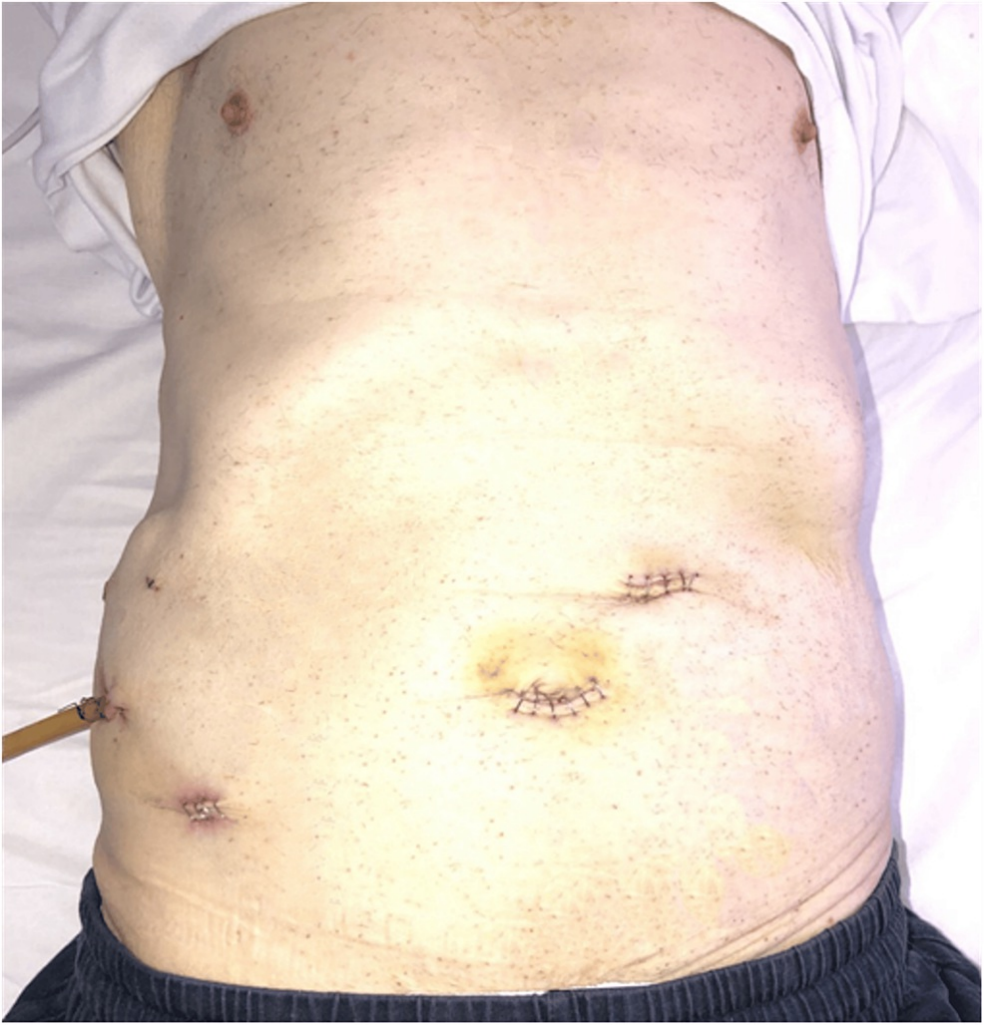
Postoperative day seven: position of the laparoscopic abdominal port (five-port technique).

**Figure 2:**
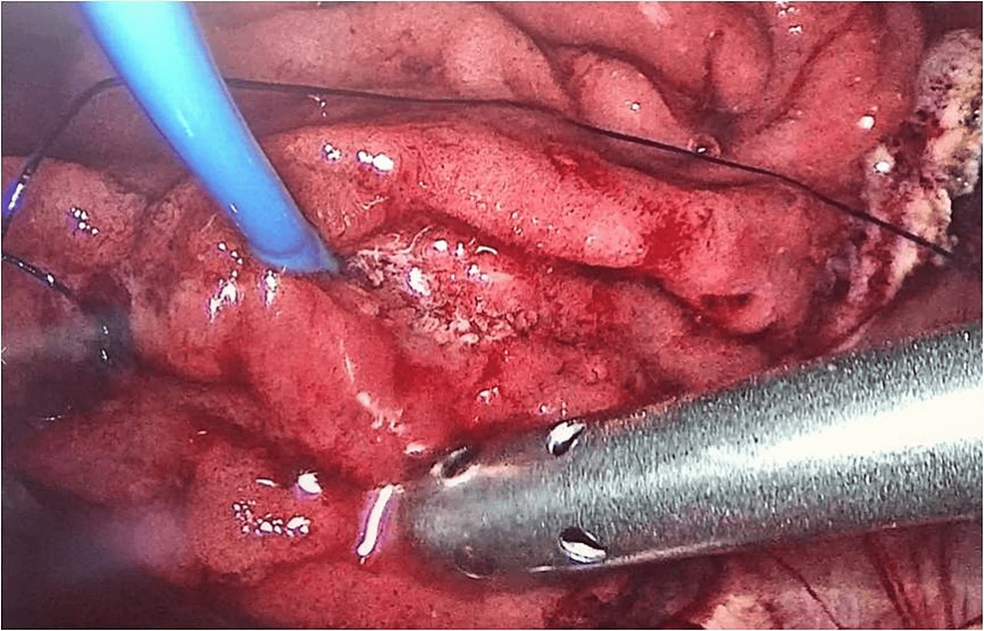
An inserted pigtail through the cystic duct to the ampulla of Vater highlighting the periampullary tumor.

**Figure 3:**
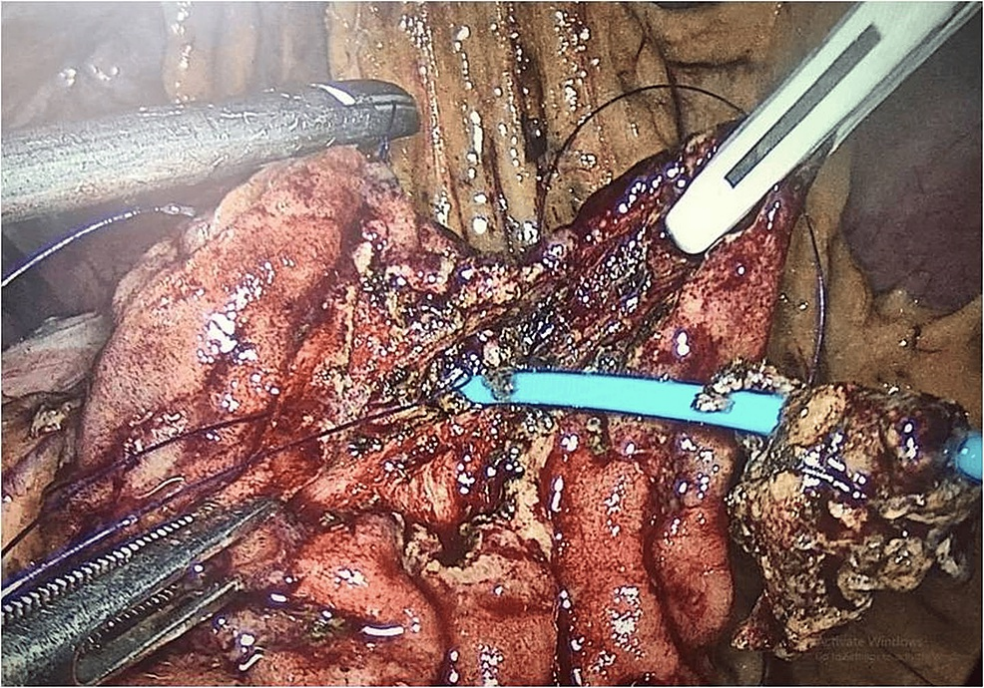
Roundly dissected ampullary tumor upon pigtail using monopolar hook cautery.

The patient was positioned supine under general anesthesia and five ports were inserted (Figure 1). A longitudinal duodenotomy of 3-4 cm was performed on the antimesenteric region of the second portion of the duodenum, as the duodenum was first kocherized. The cystic duct was then dissected and a pigtail was inserted reaching the ampulla of Vater (Figure 2). The resection of the ampullary tumor was performed using hook monopolar cautery (Figure 3). The duct was sutured to the surrounding mucosa of the duodenum from 2 until 9 o’clock position to protect the pancreatic duct. The pigtail was subsequently replaced by a plastic stent. The duodenotomy was closed in two layers transversely with PDS 3-0 sutures. A cholecystectomy was also performed. The operation lasted 210 minutes, and the estimated blood loss was minimal.

**Figure 4:**
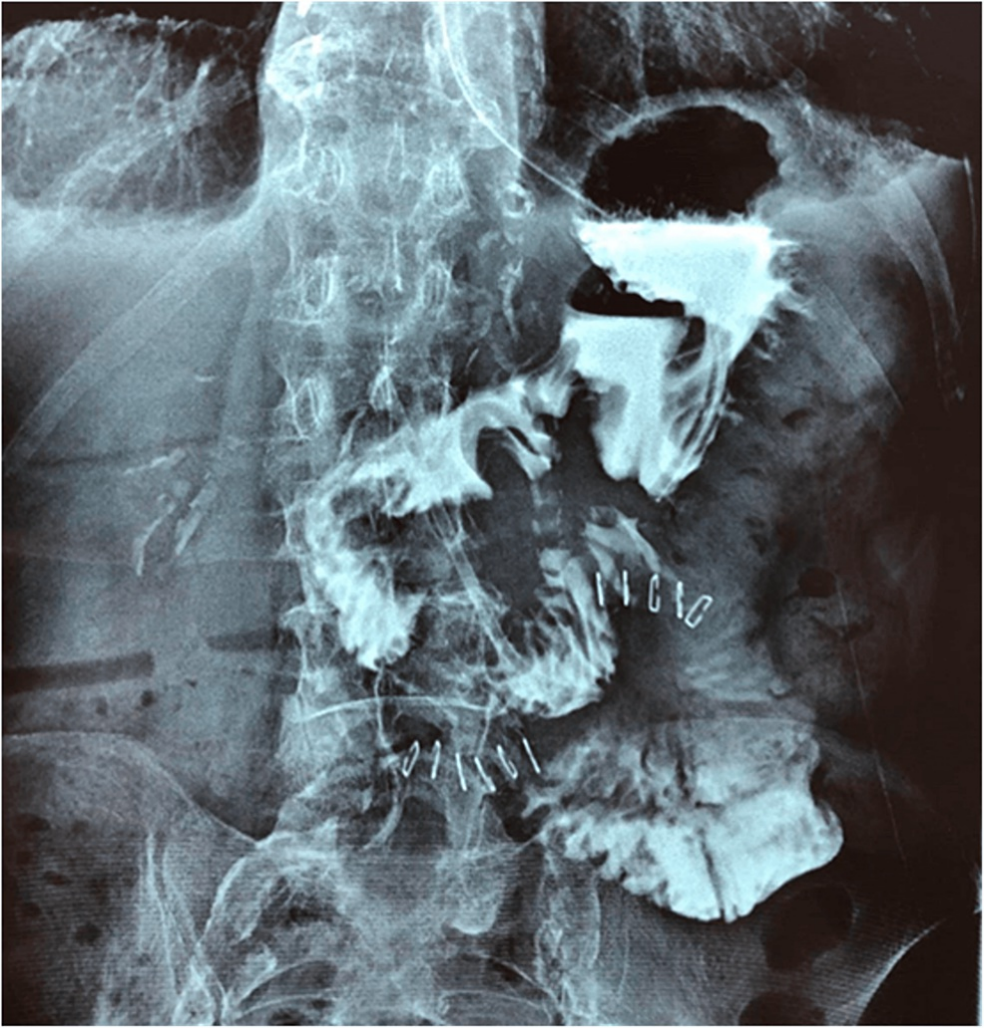
Upper gastrointestinal series on postoperative day five reveals no leakage or stenosis of the duodenum.

The patient remained in the hospital for 11 postoperative days due to SARS-CoV-2 infection. On postoperative day five, an upper gastrointestinal series showed no leakage or outflow obstruction (Figure 4). On the same day, the patient was started on clear fluids. There was no need for total parenteral nutrition, and surgical complications did not occur. The patient received antibiotic treatment until his discharge and a course of analgesics for seven days.

The tumor was completely resected, and the final pathology revealed a 1 cm adenoma of the ampulla of Vater of high-grade dysplasia (carcinoma in situ, Tis), with negative margins (R0). The patient was healthy at the four-month follow-up.

## Discussion

Endoscopic resection has been popularized recently for adenomas or early lesions. It is a method with minimal complications, including pancreatitis, bleeding, perforation, and stenosis. It has an overall morbidity of 15% and mortality of up to 0.3% [3]; however, it is not always possible or preferable owing to increased rates of local recurrence of up to 33% and excision failure in larger tumors over 2 cm. Indications for leading to surgical resection (PD or TA) include large tumor size, unsuccessful previous endoscopic resection, recurrence, extension of the neoplasm to the bile or pancreatic duct, or stricture of the ampulla [4].

PD is a demanding operation with significant morbidity, mortality rates of up to 2.6%, and complications including diabetes mellitus. TA, however, has fewer and less significant complications, such as pancreatic or bile leak/abscess, duodenal stenosis or fistulae, acute pancreatitis, and cholangitis [5]. It also shows excellent perioperative and long-term outcomes, with a mortality of less than 1%. TA could arguably become the gold standard for benign, premalignant tumors and early cancer (Tis) of the ampulla of Vater that are either too large for EP or where the PD risk/benefit ratio is unacceptable. Data regarding TA in T1 or even T2 tumors is still pending (ESAP study), but several practitioners prefer it for low-risk tumors smaller than 3 cm, especially for low-grade tumors, or for patients unfit for PD. Additional lymphadenectomy has been argued for tumors carrying a higher risk of lymph node metastasis [6].

Laparoscopic or robotic-assisted TA has lower rates of perioperative blood loss, shorter hospitalization, fewer perioperative complications, and low recurrence rates [7]. The laparoscopic TA might be challenging, but it bears all the advantages of the robotic method, only with a considerably lower cost.

## Conclusions

TA is a surgical option for ampullary tumors such as adenoma, high-grade dysplasia, and even early ampullary cancer (T1) which cannot be excised endoscopically or ampullary cancer >T2 that has no indications for pancreatoduodenectomy, which, if done laparoscopically, results in excellent oncological results with minimal morbidity and quick patient recovery. Laparoscopic transduodenal sphincteroplasty is challenging and requires surgical skills in minimally invasive procedures.
